# In Vitro Effect of Enzymes and Human Milk Oligosaccharides on FODMAP Digestion and Fecal Microbiota Composition

**DOI:** 10.3390/nu15071637

**Published:** 2023-03-28

**Authors:** Van T. Pham, Robert E. Steinert, Cindy Duysburgh, Jonas Ghyselinck, Massimo Marzorati, Peter J. T. Dekker

**Affiliations:** 1 DSM Nutritional Products, Wurmisweg 576, 4303 Kaiseraugst, Switzerland; 2 ProDigest, Technologiepark 82, Zwijnaarde, 9052 Ghent, Belgium; 3 Center for Microbial Ecology and Technology, Faculty of Bioscience Engineering, University of Ghent, Coupure Links 653, 9000 Ghent, Belgium; 4 DSM Food & Beverage, Center for Food Innovation, Fleminglaan 1, 2613 AX Delft, The Netherlands

**Keywords:** α-galactosidase, β-galactosidase, digestive enzymes, FODMAP, human milk oligosaccharide, inflammatory bowel syndrome, invertase

## Abstract

Fermentable oligosaccharides, disaccharides, monosaccharides, and polyols (FODMAPs) cause intestinal discomfort in patients with irritable bowel syndrome (IBS). An enzyme mix (2500 SU invertase, 2400 GalU α-galactosidase, 10,000 ALU β-galactosidase) optimized for FODMAP digestion, and/or human milk oligosaccharides (HMO) (2′-FL, DFL, and LNnT), were evaluated for effects on microbial community activity and composition in short-term colonic incubations using the fecal microbiota of four patients with IBS-D symptoms under the following test conditions: (i) FODMAP, (ii) pre-digested (with enzyme mix) FODMAP, (iii) FODMAP + HMO, and (iv) pre-digested FODMAP + HMO. Pre-digested FODMAP reduced short-chain fatty acid (SCFA) production versus FODMAP; HMO restored this. A 10-day experiment with the simulator of the human intestinal microbial ecosystem (SHIME^®^), using fecal samples from two patients with IBS-D, further evaluated these findings. FODMAP resulted in decreased microbial diversity versus blank. Pre-digestion with the enzyme mix restored microbial diversity, improved FODMAP digestibility, and reduced gas pressure versus undigested FODMAP; however, SCFA production decreased. HMO restored SCFA production along with an increase in gas pressure and increased abundance of *Lachnospiraceae*. When used in combination, the FODMAP enzyme mix and HMO may resolve FODMAP-related IBS symptoms while maintaining a healthy gut microbiome via prebiotic activity.

## 1. Introduction

Fermentable oligosaccharides, disaccharides, monosaccharides, and polyols (FODMAPs) are short-chain carbohydrates that include fructose, lactose, fructan, galactan, and polyols [1]. FODMAPs are poorly absorbed in the small intestine, are osmotically active, and are rapidly fermented by bacteria in the colon [2]. This rapid colonic fermentation results in increased gas production [3,4] which sometimes may lead to abdominal distension and diffuse abdominal pain.

Irritable bowel syndrome (IBS) is a gastrointestinal disorder in which patients experience recurrent abdominal pain and abnormalities in stool form or frequency [4]. Approximately 5% to 10% of the general population suffer from IBS [5]. Patients with IBS may experience an exacerbation of symptoms when consuming foods rich in FODMAPs; it is suggested that visceral hypersensitivity to luminal distension is likely responsible for carbohydrate-related symptoms in these patients [6,7]. Many studies have reported that a low-FODMAP diet can alleviate some symptoms of IBS (reviewed in [8]). For example, it has been reported to reduce symptoms of abdominal pain, bloating, constipation, diarrhea, abdominal distention, and flatulence that patients with IBS experience when consuming a normal diet and can improve the quality of life score in some studies. The American College of Gastroenterology clinical guidelines for the management of IBS recommends a low-FODMAP diet on a limited basis to improve global symptoms [9]. However, concerns about nutritional adequacy, cost, and difficulty with learning and maintaining the diet have been raised. The diet begins with a global restriction of FODMAP consumption over a period of 4 to 8 weeks, followed by gradual reintroduction of FODMAP foods [10]. A qualified nutritionist should be involved as adequate micronutrient intake may be challenging [11]. Given the importance of professional advice when following this diet [12], it may not be suitable for or accessible to all patients with IBS. Additionally, a low-FODMAP diet may be difficult to adhere to [13,14] and can be expensive [15], time consuming, and socially restrictive [14].

Another potential issue with the long-term implementation of a low-FODMAP diet is the fact that healthy plant foods that act as prebiotics and support a healthy gut microbiota are restricted [16]. Therefore, a low-FODMAP diet may affect the composition of the gut microbiota. Studies have shown that total bacterial abundance and the *Bifidobacterium* population both decrease while the ratio of *Firmicutes* to *Bacteroidetes* increases after only 2–3 weeks of a low-FODMAP diet [16,17]. These results indicate that caution should be taken when recommending a low-FODMAP diet in the long-term [16].

Digestive enzymes catalyze the breakdown of complex macromolecules and thus may be beneficial to human health via their ability to aid in digestion. For example, recent evidence suggests that gluten-degrading enzymes may aid patients with celiac disease [18]. Along the same line, digestive enzymes that catalyze the breakdown of FODMAPs may be a useful tool for patients with IBS; however, the number of such products that are currently available is limited. However, additional enzyme supplementation to aid in FODMAP digestion may limit the amount of prebiotic fiber reaching the colon over time. In turn, this may result in negative effects on the activity and composition of the gut microbiota. To compensate for this, co-supplementation with a non-FODMAP prebiotic substance should be considered. 

Human milk oligosaccharides (HMOs) are a group of complex and diverse glycans that are present in human breast milk and act as prebiotics [19]. While HMOs play a critical role in shaping the gut microbiota of infants due to their bifidogenic effect, it is also suggested that they have a beneficial effect on the adult gut microbiome [20]. These beneficial effects include an increase in bifidobacteria, increased short-chain fatty acid (SCFA) production, reduced intestinal permeability, and an immunomodulatory effect. The two most common HMOs are 2′-O-fucosyllactose and lacto-N-neotetraose (LNnT). When used as a standalone treatment in patients with IBS, they have been shown to improve IBS symptoms and quality of life, and to increase beneficial non-gas producing *Bifidobacterium* spp. without causing gastrointestinal symptoms [21,22]. 

Both FODMAP-digesting enzymes and HMOs may alleviate IBS symptoms through different mechanisms, suggesting that the two strategies may be complementary. Therefore, we hypothesized that a combination of enzymes to digest the three most relevant FODMAPs (fructan, galactan, and lactose) would lead to a reduction of excessive bacterial growth in the colon and may reduce symptoms in IBS patients. Combining such an enzyme blend with HMO may stimulate the bifidogenic population and related SCFA production, thereby restoring a healthy gut function in IBS patients without causing intestinal discomfort such as bloating. This in vitro study aimed to evaluate the effects of a digestive enzyme and/or HMO supplementation on the microbial community activity and microbial community composition using in vitro models of human digestion of FODMAPs.

## 2. Materials and Methods

The design of this study, which took place in three sequential phases, included an upper intestinal tract simulation (phase I), short-term colonic batch studies (phase II), and a QuintSHIME^®^ experiment (phase III). In the first phase, an enzyme mix was optimized for the purpose of aiding in the digestion of FODMAPs, which was evaluated with or without HMO co-supplementation in the second phase using short-term colonic simulations inoculated with fecal microbiota samples from four patients with IBS with diarrhea (IBS-D). In the third phase, the enzyme mix was tested with or without HMO co-supplementation using a simulator of the human intestinal microbial ecosystem (SHIME^®^) (ProDigest, Gent, Belgium). The effects of supplementation in the presence of FODMAPs on the microbial community activity and composition were determined, again using fecal samples from two patients with IBS-D.

### 2.1. Upper Gastrointestinal Tract Simulation

Simulation of the upper gastrointestinal tract (GIT) was accomplished by utilizing an adapted SHIME^®^ system that represents the conditions of the stomach and small intestine within the same reactor over time. This study mimicked fed conditions in the stomach using a gastric suspension (gastric phase) and in the small intestine by adding a standardized enzyme mix and bile liquid (small intestine phase). In general, the conditions used were based on the InfoGest Consensus Method [23] with some modifications, such as including gradual pH changes, to best represent conditions in vivo. For the gastric phase, gastric juice containing a standardized mix with pepsin (standardized to an activity of 4000 U/mL) (Chem Lab, Zedelgem, Belgium), lecithin (13.5 g/L) (Carl Roth GmbH + Co.KG, Germany), sodium chloride (50 mM), potassium chloride (7 mM), and mucin were added to the chamber which was incubated for 2 h at 37 °C with stirring. Over time, the pH decreased from 5.5 to 2.0 in a sigmoidal fashion. For the small intestine phase, the pH was increased from 2.0 to 5.5 in the first 5 min, followed by a gradual increase from 5.5 to 7.0 during the 3 h incubation at 37 °C. Pancreatic juices containing NaHCO_3_ (7.7 g/L), ox gall (15 g/L), pancreatin (10 g/L), 2.15 mL trypsin (10 g/L), and 2.7 mL chymotrypsin (10 g/L) (Difco, Bierbeek, Belgium), were added at the start of the small intestine phase.

Test substrates were used to evaluate the digestion capability of the FODMAP enzymes and enzyme mixtures. These included short-chain fructo-oligosaccharides (FOS) (Actilight FOS 950P; Béghin Meiji, Marckolsheim, France; 2 g per reactor) to represent fructans, lactose (VWR, Radnor, PA, USA; 5 g), and a mixture of stachyose (Acros Organics, Geel, Belgium; 0.5 g) and raffinose (Alfa Aesar, Haverhill, MA, USA; 0.5 g) to represent galactans.

Each FODMAP enzyme was added at a low, medium, or high concentration to determine the optimal conditions for sugar digestion. The test enzymes included were invertase (Maxinvert 200,000 MG (DSM, Heerlen, The Netherlands); 500 SU [2.5 mg per reactor], 2500 SU [12.5 mg], 10,000 [50 mg]) to digest fructans (FOS), α-galactosidase (Fibractase, (Intoleran, Friesland, The Netherlands); 600 GalU [150 mg], 1200 GalU [300 mg], 2400 GalU [600 mg]) to digest galactans (stachyose and raffinose), and β-galactosidase (Tolerase L, (DSM, Heerlen, The Netherlands); 1000 ALU [10 mg], 2500 ALU [25 mg], 10,000 ALU [100 mg]) to digest lactose. After the optimal concentration of each FODMAP enzyme was determined, a mixture was used to determine the effects on the digestion of a combination of all four sugars, or a 1:1:1:1 mixture of three complex foods (17.85 g each per reactor) including red kidney beans, industrial white bread, skim milk, and water to simulate galactans, fructans, and lactose-containing foods, respectively.

Each reactor had a total volume of 100 mL, except when complex foods were used, and each reactor had a total volume of 117.85 mL due to the additional volume of the test substrates. Test substrates were added to the reactors at the start of the gastric phase prior to sampling; the test enzymes were added at the start of the gastric phase immediately after sampling. Reactors were sampled at the start of the gastric phase and at the end of the small intestine phase. Blank controls (no enzyme) were used to quantify the effects of the test enzymes. All experiments were performed in a single repetition and formed part of the basis for the conditions used in the 10-day SHIME^®^ experiment. 

### 2.2. Carbohydrate Quantification and FOS Profiling

The concentration of lactose, glucose, galactose, fructose, stachyose, and raffinose was assessed using high performance anion exchange chromatography with pulsed amperometric detection (HPAEC-PAD). Upon collection, samples were diluted to obtain a concentration between 0 and 200 μM for each of the sugars present. Dilutions were prepared in water, with a final 1:1 dilution in acetonitrile to remove complex polymers from the samples. An internal standard (rhamnose) was included in the samples, reaching a final concentration of 25 μM. Samples were centrifuged and filtered prior to measurement by HPAEC-PAD (Dionex, Sunnyvale, CA, USA) under highly alkaline conditions. A calibration curve was generated for each sugar. The concentration of each sugar was calculated based on its calibration curve. Total monosaccharide content (%) was determined by dividing the total amount of monosaccharides by the total sugar content. Samples for qualitative profiling of FOS were centrifuged, filtered, then measured by HPAEC-PAD. Samples were collected at the start of the gastric incubation (before addition of the test enzymes) and at the end of the small intestinal incubation.

### 2.3. Short-Term Colonic Incubations

The colon microbiota from four fecal donors with IBS-D was screened for suitability for use in a 10-day SHIME^®^ experiment using short-term single-stage colonic incubations. Fecal material was collected from each donor and suspensions were prepared and mixed with an optimized cryoprotectant; aliquots were flash frozen and stored at −80 °C until needed. Fecal samples were thawed immediately prior to being added to the reactors. 

Short-term colonic incubations were performed as previously described [24]. Briefly, sugar-depleted nutritional medium which contained the basal nutrients of the colon, was used to fill each individual colonic reactor. Next, each reactor was given additional medium (blank) or one of the following test substrates: 100% FODMAP (0.86 g/L FOS + 0.21 g/L stachyose + 0.21 g/L raffinose + 1.71 g/L lactose), 100% FODMAP + HMO (Glycom A/S, Syddanmark, Denmark) (FODMAP mixture + 2.4 g/L 2′fucosyllactose [2′-FL]/difucosyllactose [DFL] mixture + 0.6 g/L LNnT), pre-digested FODMAP (0.05 g/L stachyose + 0.10 g/L raffinose + 0.11 g/L lactose), and pre-digested FODMAP + HMO (pre-digested FODMAP + 2.4 g/L 2′-FL/DFL + 0.6 g/L LNnT). The substrate concentrations in the pre-digested arms were calculated based on digestion profiles of each enzyme from phase I. Next, reactors were inoculated with fecal material from one of four donors with IBS (five reactors per donor, one control reactor [blank] and four test substrate reactors). The reactors were incubated for a total of 48 h at 37 °C with continuous shaking (90 rpm) under anaerobic conditions. Each reactor was fully independent. pH, gas pressure, gas composition, and SCFA levels were measured at 0, 6, 24, and 48 h; microbial community composition was evaluated at 48 h. 

### 2.4. QuintSHIME^®^ Model

The SHIME^®^ model, which was first described by Molly et al. in 1993 [25], allows culturing of the complex gut microbiota over a longer period under representative conditions of the different intestinal regions. The traditional setup consists of a succession of five reactors simulating different regions of the human GIT (stomach, small intestine, and the ascending, transverse, and descending colon). Retention time, pH, temperature settings, and reactor feed composition were previously described by Possemiers et al. [26]. This study used a variation of the traditional SHIME^®^ setup called the QuintSHIME^®^ configuration which allows the comparison of five different conditions in parallel. Fecal microbiota from two different donors with IBS were evaluated in this model, meaning that two identical, other than the fecal microbiota donor, but separate QuintSHIME^®^ configurations were used. These configurations consisted of a combined stomach and small intestine reactor vessel (upper GIT) and a single colon reactor vessel to simulate the transverse colon (TC). The stomach/small intestine compartment operated according to the fill-and-draw principle. Peristaltic pumps added 140 mL of SHIME^®^ feed (pH 3.0) to simulate the stomach (1.5 h incubation) then pancreatic and bile liquid (60 mL) was added and the pH adjusted to 6 to simulate the small intestine (1.5 h incubation). The intestinal suspension was then pumped into the TC reactor (pH 6.15–6.4; retention time, 20 h; volume, 500 mL). On day 0, the colon reactors were inoculated with a fecal sample from one of two fecal donors with IBS. The microbiota was allowed to grow and colonize in the reactor for 1 day to support the maximum diversity of the gut microbiota originally present in the fecal sample. This was followed by a 10-day treatment period in which the QuintSHIME^®^ reactor was fed three times daily with sugar-depleted SHIME^®^ nutritional medium supplemented with test products. 

Test conditions were blank, FODMAP, pre-digested FODMAP, FODMAP + HMO, and pre-digested FODMAP + HMO.

Samples were collected on day 9 for gas production analysis using the short-term colonic simulation model as described above. Samples were collected to determine SCFA levels and microbial community composition by quantitative 16S-targeted Illumina sequencing on days 9 and 10.

### 2.5. Microbial Community Activity 

pH was measured at the indicated timepoints using a Senseline F410 pH meter (ProSense, Oosterhout, The Netherlands) and a hand-held pressure indicator (CPH6200; Wika, Echt, The Netherlands) was used to measure gas pressure. Gas composition was evaluated using a compact gas chromatograph (Global Analyser Solutions, Breda, The Netherlands) which was equipped with a Molsieve 5A pre-column and Porabond column (for CH_4_, and H_2_), a Rt-Q-bond pre-column and column (for CO_2_ and H_2_S), and a thermal conductivity detector. SCFAs (acetate, propionate, butyrate) were measured as previously described [27]. Samples were collected from the TC compartment on days 9 and 10 and were considered replicates.

### 2.6. Microbial Community Composition Analysis by qPCR

Luminal samples were subjected to quantitative polymerase chain reaction (qPCR) to quantify specific populations of the simulated human gut microbiota. DNA isolation and qPCR analyses were performed as described by Van den Abbeele et al. [28]. Briefly, a StepOnePlus™ real-time PCR system (Applied Biosystems, Foster City, CA, USA), was used to quantify four taxonomic groups of interest, i.e., *Bifidobacterium* spp. [28], Bacteroidetes [28], Firmicutes [28], and *Faecalibacterium prausnitzii* [29]. The Firmicutes/Bacteroidetes ratio was calculated by dividing the abundance of Firmicutes by the abundance of Bacteroidetes for each sample.

### 2.7. Microbial Community Composition Analysis by Quantitative 16S-Targeted Sequencing and Flow Cytometry

For the QuintSHIME^®^ experiments, microbial community composition was assessed on days 9 and 10 using samples taken from the TC compartment; the two samples were considered as replicates for analysis. A blank sample for each donor was analyzed and used as a reference. Next-generation 16S rRNA gene amplicon sequencing of the V3–V4 region was performed by LGC Genomics GmbH (Berlin, Germany). An Illumina MiSeq platform with v3 chemistry was used for library preparation and sequencing. The previously described 341F (5′-CCTACGGGNGGCWGCAG-3′) and 785R (5′-GACTACHVGGGTATCTAAKCC-3′) primers were used [30] with a reverse primer that was adapted to increase coverage. PCR for quality control was performed using Taq DNA Polymerase with the Fermentas PCR Kit according to the manufacturers’ instructions (ThermoFisher, Waltham, MA, USA). Gel electrophoresis (2% [*w*/*v*] agarose gel, 30 min, 100 V) was employed to verify DNA quality. Amplicon data were analyzed as previously described [31]. Heat maps were used to visualize the data output; data were separated by donor (donor 3 and donor 4). Heatmaps were generated using the MicrobiomeAnalyst in the Marker Data Profiling software (https://www.microbiomeanalyst.ca/MicrobiomeAnalyst/home.xhtml, accessed on 10 June 2022) with a Pearson distance measure and Ward clustering algorithm.

Bacterial cell densities were measured using a BD FacsVerse flow cytometer (BD Biosciences, Franklin Lakes, NJ, USA) and live cells were distinguished from damaged cells using SYBR Green (Thermo Fisher, Merelbeke, Belgium)/propidium iodide (Merck KGaA, Darmstadt, Germany) staining, as previously described [32]. Proportional values obtained with Illumina sequencing were converted into absolute quantities by correcting with live bacterial cell counts. Bacterial cells were separated from medium debris and signal noise by applying a threshold level of 200 on the SYTO channel.

### 2.8. Statistical Analysis

No statistical tests were applied to data from experiments that were performed in a single repetition. For the short-term colonic incubations, data are shown as averages across the donors and statistical comparisons between the different test conditions have been performed using a two-tailed paired *t*-test. A two-tailed homoscedastic t-test was used for the statistical comparison of the different test conditions for the QuintSHIME^®^ portion of the study. For microbial community composition, data were log-transformed prior to performing statistical analyses to achieve a normal distribution. A *p*-value of <0.05 was considered statistically significant.

## 3. Results

### 3.1. Upper GIT Simulation

FOS, stachyose, raffinose, and lactose were degraded by the test enzymes in a dose dependent fashion (Table 1) in the upper GIT simulation system. The maximum digestion of FOS (100%) was achieved with 2500 SU and 10,000 SU of invertase, and of stachyose (76%) and raffinose (52%) with 2400 GalU α-galactosidase, and of lactose (94%) with 10,000 ALU β-galactosidase. Optimal doses of invertase, α-galactosidase, and β-galactosidase of 2500 SU (12.5 mg), 2400 GalU (600 mg), and 10,000 ALU (100 mg), respectively, were selected for further testing as an enzyme mix (optimized enzyme mix).

**Figure 1:**
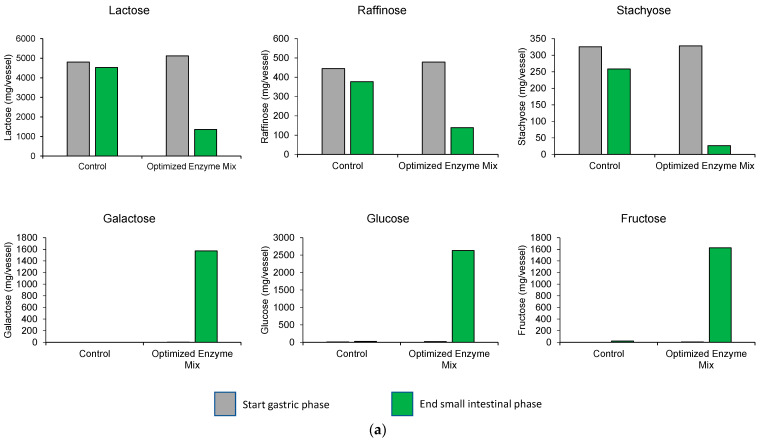

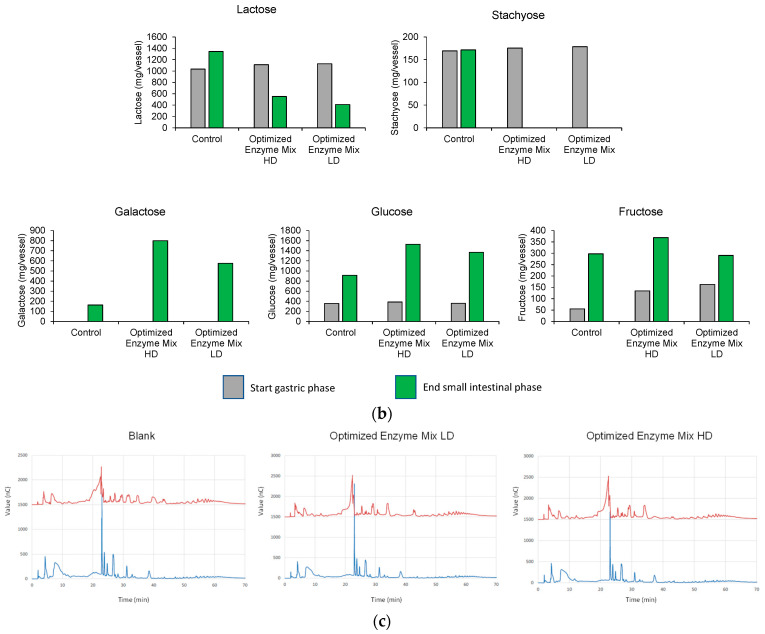
Digestion characteristics of the individual FODMAP components in upper GIT simulations with synthetic food substrates (**a**) and complex food substrates (**b**) and qualitative FOS profiling during upper GIT passage (complex food substrates) (**c**). During upper GIT simulations, levels of various products of FODMAP digestion were determined at the start of the gastric phase (gray bars) and at the end of the small intestinal phase (green bars) with no enzyme (blank) and those supplemented with the optimized enzyme mix (12.5 mg invertase [2500 SU], 100 mg β-galatosidase [10,000 ALU], 600 mg α-galactosidase [2400 GalU]). The synthetic food substrates included 2 g FOS, 5 g lactose, 0.5 g stachyose, and 0.5 g raffinose and the complex food substrates included 17.5 g each of industrial white bread, red kidney beans, and skim milk. For the qualitative FOS profiling, the blue line represents the qualitative FOS profiling determined at the start of the gastric phase and the red line represents the same at the end of the small intestinal phase. GIT = gastrointestinal; FODMAP = fermentable oligosaccharides, disaccharides, monosaccharides, and polyols; FOS = fructooligosaccharide; HD = high dose; LD = low dose.

Upper GIT simulation using synthetic food substrates (2 g FOS, 5 g lactose, 0.5 g stachyose, and 0.5 g raffinose) was conducted with no enzymes (blank) or the optimized enzyme mix. At the end of the small intestine incubation, levels of lactose, raffinose, and stachyose were reduced compared with those at the beginning of the stomach digestion in chambers with optimized enzyme mix, while little change was observed in reactors with no optimized enzyme mix (blank) (Figure 1a). Additionally, monosaccharide levels (galactose, glucose, and fructose) were very low at the start of the GIT simulation but were increased over time with the optimized enzyme mix, indicating that carbohydrate digestion took place; levels remained low in the blank chambers. Overall, these results demonstrated that the optimized enzyme mix degraded the FODMAP substrates while no digestion could be detected in the blank control during upper GIT transit.

Next, the optimized enzyme mix was evaluated in the upper GIT simulation using complex food substrates (17.5 g each: industrial white bread, red kidney beans, and skim milk). Two doses of the optimized enzyme mix were tested, the standard dose used in the upper GIT simulation with synthetic food substrates (12.5 mg invertase [2500 SU], 100 mg β-galactosidase [10,000 ALU], 600 mg α-galactosidase [2400 GalU]), or a low dose which was 25% of the standard dose (3.125 mg invertase [625 SU], 25 mg β-galactosidase [2500 ALU], 150 mg α-galactosidase [600 GalU]). In general, similar amounts of digestion were observed whether the standard or low dose of the optimized enzyme mix was used (Figure 1b). The optimized enzyme mix enabled lactose digestion and carbohydrate (galactan and fructan) digestion, as demonstrated by an increase in monosaccharides. Qualitative FOS profiling demonstrating that the optimized enzyme mix degraded FOS to a greater extent compared with the blank control is shown in Figure 1c, and indicated by the presence of a higher concentration of peaks at shorter retention times (corresponding to short-chain carbohydrate fractions) compared to the blank control. Raffinose was not detected in any of the samples with complex food substrates, making it likely that the amount of raffinose initially present in each of the foods was very low. 

### 3.2. Short-Term Colonic Incubations

Short-term colonic incubations using the fecal microbiota from four individual donors with IBS-D were conducted to determine the effects of the optimized enzyme mix and/or HMO on gas pressure and gas composition, SCFA levels, and the levels of select members of the gut microbiome when FODMAP is provided as a food source.

**Figure 2:**
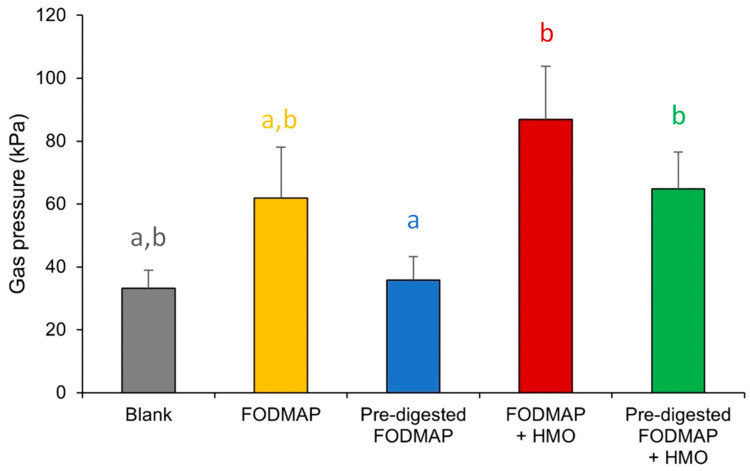
Changes in total gas pressure in short-term colonic batch experiments (48 h). Average ± SEM is shown across donors. Different letters represent statistically significant differences between the different test conditions (*p* < 0.05). The test substrates were as follows: FODMAP (0.86 g/L FOS + 0.21 g/L stachyose + 0.21 g/L raffinose + 1.71 g/L lactose), pre-digested FODMAP (0.05 g/L stachyose + 0.10 g/L raffinose + 0.11 g/L lactose), FODMAP + HMO (FODMAP composition + 2.4 g/L 2′FL/DFL + 0.6 g/L lacto-N-neotetraose), pre-digested FODMAP + HMO (pre-digested FODMAP composition + 2.4 g/L 2′FL/DFL + 0.6 g/L lacto-N-neotetraose). 2′FL = 2′fucosyllactose; DFL = difucosyllactose; FODMAP = fermentable oligosaccharides, disaccharides, monosaccharides, and polyols; HMO = human milk oligosaccharide.

FODMAP alone increased the overall gas pressure compared with the blank, though not reaching statistical significance across the donors, and the addition of HMO resulted in a further increase for most donors. Importantly, when FODMAP was pre-digested using the optimized enzyme mix, gas pressure was reduced to levels similar to the blank, indicating reduced microbial activity; again, when HMO was added, gas pressure increased, indicating increased microbial activity (Figure 2). No notable differences were observed in gas composition between treatments (data not shown).

**Figure 3:**
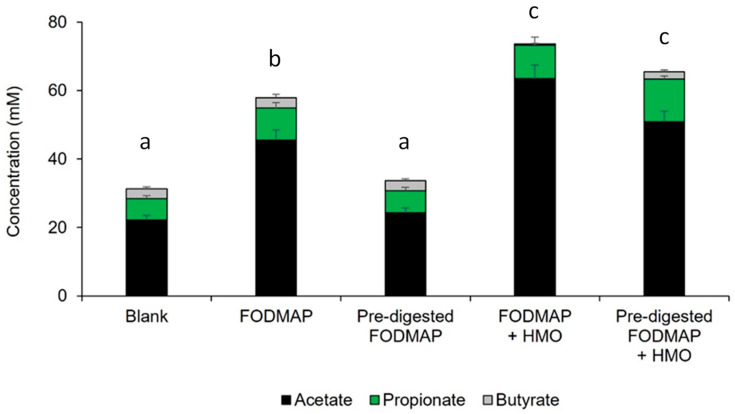
Changes in total SCFA, propionate, acetate, and butyrate in short-term colonic batch experiments (48 h). Total SCFA, propionate, acetate, and butyrate levels during different time intervals of colonic incubation are shown. Average ± SEM is shown across donors. Different letters represent statistically significant differences between the different test conditions with respect to total SCFA (*p* < 0.05). The test substrates were as follows: FODMAP (0.86 g/L FOS + 0.21 g/L stachyose + 0.21 g/L raffinose + 1.71 g/L lactose), pre-digested FODMAP (0.05 g/L stachyose + 0.10 g/L raffinose + 0.11 g/L lactose), FODMAP + HMO (FODMAP composition + 2.4 g/L 2′FL/DFL + 0.6 g/L lacto-N-neotetraose), pre-digested FODMAP + HMO (pre-digested FODMAP composition + 2.4 g/L 2′FL/DFL + 0.6 g/L lacto-N-neotetraose). 2′FL = 2′fucosyllactose; DFL = difucosyllactose; FODMAP = fermentable oligosaccharides, disaccharides, monosaccharides, and polyols; HMO = human milk oligosaccharide; SCFA = short-chain fatty acid.

Regarding total SCFAs, pre-digestion significantly decreased the total SCFA production relative to FODMAP. However, when HMOs were added, the total SCFA level recovered to levels similar to FODMAP (without pre-digestion) (Figure 3). A similar pattern was observed for individual SCFAs of propionate and acetate. The pattern for butyrate was different. For all donors combined, the butyrate level between FODMAP and pre-digested FODMAP was similar; when HMOs were added to FODMAP, the level of butyrate decreased, while pre-digested FODMAP + HMO had a butyrate level similar to FODMAP and pre-digested FODMAP (without HMO).

**Figure 4:**
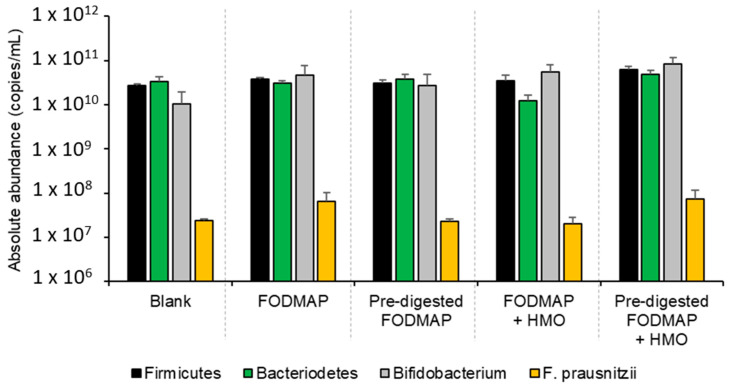
Absolute abundance of Firmicutes, Bacteroidetes, *Bifidobacterium*, and *Faecalibacterium prausnitzii* in short-term colonic batch experiments (48 h). Average ± SEM is shown across donors. The test substrates were as follows: FODMAP (0.86 g/L FOS + 0.21 g/L stachyose + 0.21 g/L raffinose + 1.71 g/L lactose), pre-digested FODMAP (0.05 g/L stachyose + 0.10 g/L raffinose + 0.11 g/L lactose), FODMAP + HMO (FODMAP composition + 2.4 g/L 2′FL/DFL + 0.6 g/L lacto-N-neotetraose), pre-digested FODMAP + HMO (pre-digested FODMAP composition + 2.4 g/L 2′FL/DFL + 0.6 g/L lacto-N-neotetraose). 2′FL = 2′fucosyllactose; DFL = difucosyllactose; FODMAP = fermentable oligosaccharides, disaccharides, monosaccharides, and polyols; HMO = human milk oligosaccharide.

Microbiome composition was also evaluated. The absolute abundance of Firmicutes, Bacteroidetes, *Bifidobacterium*, and *F. prausnitzii* was highest for the pre-digested FODMAP + HMO sample, though only reaching significance for Firmicutes (Figure 4). The Firmicutes/Bacteroidetes ratio tended to be highest for the 100% FODMAP + HMO samples, and in general, the addition of HMO to the samples resulted in an increase in the Firmicutes/Bacteroidetes ratio versus blank, regardless of whether the FODMAP was pre-digested (Figure S1).

### 3.3. QuintSHIME^®^ Model

**Figure 5:**
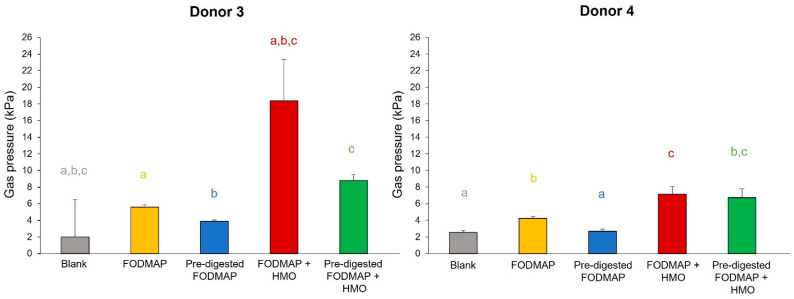
Changes in gas pressure using samples from the QuintSHIME^®^ model. Supernatants were collected from the QuintSHIME^®^ model on day 9 (treatment period) and evaluated in the short-term colonic batch model (48 h) to evaluate gas pressure (*n* = 2 for each donor). Gas pressure evaluations during different time intervals of colonic incubation are shown. Different letters represent statistically significant differences between the different test conditions (*p* < 0.05). FODMAP = fermentable oligosaccharides, disaccharides, monosaccharides, and polyols; HMO = human milk oligosaccharide; SHIME = simulator of the human intestinal microbial ecosystem.

Donors 3 and 4 were selected for use in the medium-term QuintSHIME^®^ model in which the long-term effects of repeated administration of FODMAP, pre-digested FODMAP, FODMAP + HMO, and pre-digested FODMAP + HMO on the microbial community activity and composition were assessed. Pre-digested FODMAP resulted in decreased gas pressure relative to FODMAP; pre-digested FODMAP + HMO also resulted in reduced gas pressure relative to FODMAP + HMO in both donors with the effect more pronounced for donor 3 (Figure 5). Addition of HMO in the presence of FODMAP or pre-digested FODMAP increased gas pressure compared to FODMAP and pre-digested FODMAP alone, respectively. 

**Figure 6:**
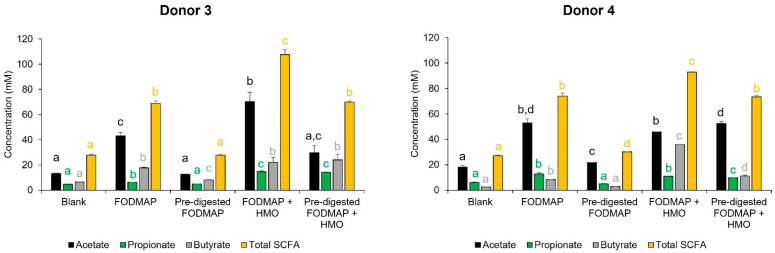
Changes in total SCFA, propionate, acetate, and butyrate in the QuintSHIME^®^ model. At the end of the treatment period (day 10), total SCFA, propionate, acetate, and butyrate production were quantified (*n* = 2 for each donor). Different letters represent statistically significant differences between the different test conditions (*p* < 0.05). FODMAP = fermentable oligosaccharides, disaccharides, monosaccharides, and polyols; HMO = human milk oligosaccharide; SCFA = short-chain fatty acid; SHIME = simulator of the human intestinal microbial ecosystem.

Changes in total SCFA were similar for both donors (Figure 6). SCFA production significantly increased relative to blank with FODMAP alone. The addition of the optimized enzyme mix reduced SCFA production to levels that were similar to blank; however, the addition of HMO to the enzyme mix resulted in SCFA levels similar to those observed for FODMAP alone. SCFA levels with FODMAP + HMO were significantly higher than with blank, FODMAP, and pre-digested FODMAP + HMO. Hence, the average SCFA production seems to follow the total amount of nutritional fiber present in the feed. There were some differences between donors when looking at individual SCFAs.

**Figure 7:**
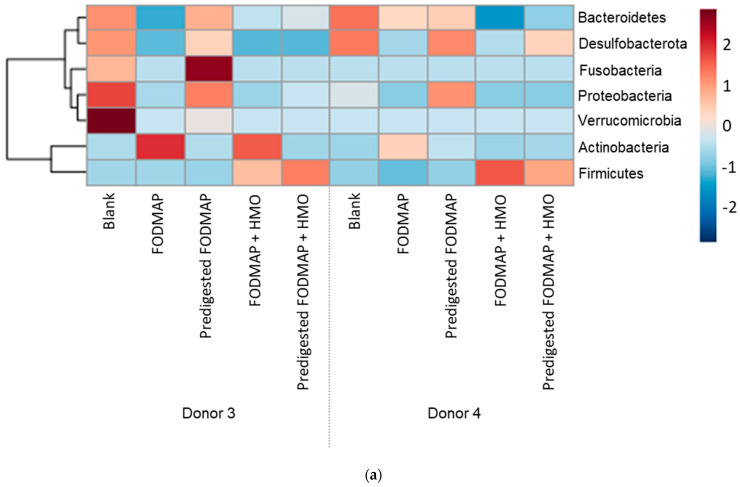

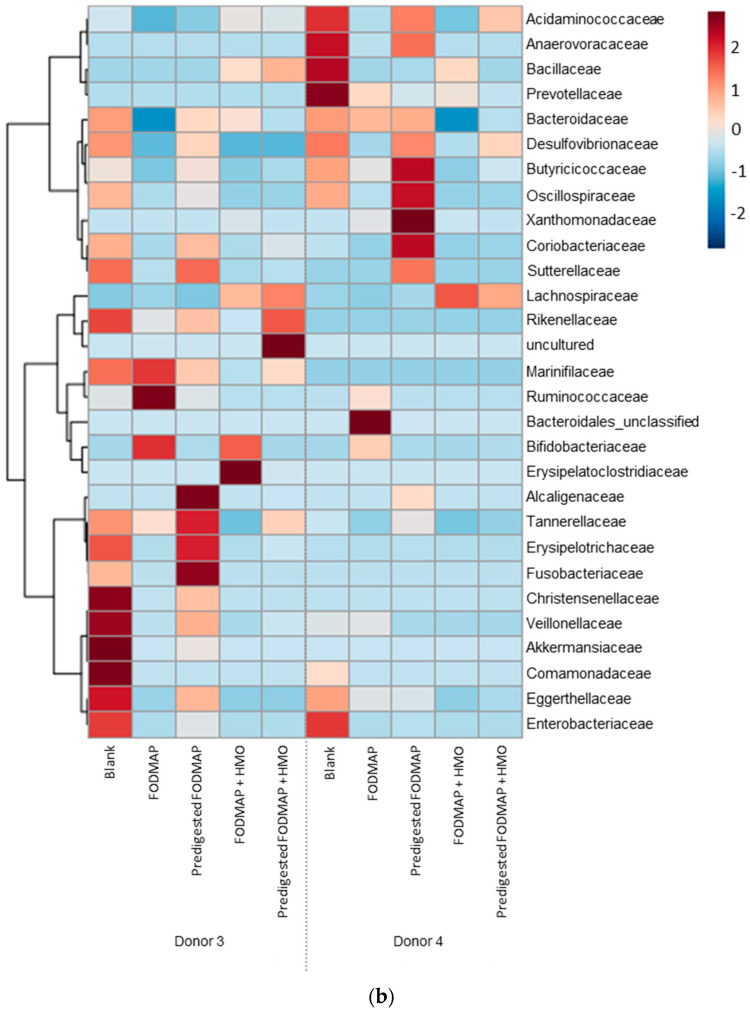
Heatmaps of microbial community composition on day 9/10 according to donor and treatment at the phylum level (**a**) and family level (**b**) in the QuintSHIME^®^ model. TC samples collected on day 9 and day 10 were considered replicates. The color of the legend represents the Z-score for each feature among the samples. Z-scores are computed on a feature-by-feature (row-by-row) basis, by subtracting the mean and then dividing by the standard deviation. This has been added to the figure caption of the revised manuscript. FODMAP = fermentable oligosaccharides, disaccharides, monosaccharides, and polyols; HMO = human milk oligosaccharide; SHIME, SHIME = simulator of the human intestinal microbial ecosystem; TC = transverse colon.

Microbial community composition data at the phylum and family levels according to donor and treatment are shown as heatmaps in Figure 7. For donor 3, the main bacteria phyla present were Actinobacteria, Bacteroidetes, Firmicutes, and Proteobacteria; members of Desulfobacteria, Fusobacteria, and Verrucomicrobia were also represented (Figure 7a and Figure S2a). Actinobacteria were higher with FODMAP, and Firmicutes were increased in the samples with pre-digested FODMAP + HMO and FODMAP + HMO versus all other conditions. The abundance of *Bacillaceae* and especially *Lachnospiraceae* was increased with pre-digested FODMAP + HMO compared with FODMAP (Figure 7b and Figure S2a). The abundance of Firmicutes increased with HMO and pre-digested FODMAP + HMO, which is mainly attributed to increased levels of the butyrate-producing *Lachnospiraceae* family and at the expense of *Ruminococcaceae* and *Desulfovibrionaceae*. HMO and pre-digested FODMAP + HMO were able to counteract the reduction in *Acidaminococcaceae* levels that was observed with FODMAP supplementation versus blank. For donor 4, the main bacteria phyla present were Actinobacteria, Bacteroidetes, Firmicutes, Proteobacteria, and Desulfobacteria (Figure 7a and Figure S2b). HMO supplementation resulted in an increase in *Lachnospiraceae* at the expense of *Bacteroidaceae* and *Desulfovibrionaceae* (Figure 7b and Figure S2b). As with donor 3, the abundance of Firmicutes increased with HMO, mainly attributed to increased levels of the *Lachnospiraceae* family. A heatmap of the microbial community composition at the genus level is shown in Figure S3. At the species level, the combination of pre-digested FODMAP + HMO resulted in an increased absolute abundance of beneficial microbes *Eubacterium ramulus*, *Clostridium bolteae*, *Blautia obeum*/*wexlerae*, and *Lachnospiraceae* spp. For both donors (Figure S3).

Microbial diversity was significantly lower compared to the blank with FODMAP supplementation; pre-digestion of the FODMAP with the optimized enzyme mix increased microbial diversity (Table 2).

## 4. Discussion

This study examined whether the combination of an enzyme mix (to aid in the digestibility of FODMAP ingredients) and HMO (to provide a prebiotic effect) may have beneficial effects in an in vitro model of IBS. The in vitro study was conducted in three phases. Upper GIT simulations were utilized to determine the optimal concentration of each enzyme included in the enzyme mix (12.5 mg invertase [2500 SU], 100 mg β-galactosidase [10,000 ALU], 600 mg α-galactosidase [2400 GalU]). The suitability of the enzyme mix to aid in the digestion of FODMAP ingredients was then tested in short-term colonic batch experiments using fecal microbiota from four individual donors with IBS-D after which two donors were selected for further evaluation in a 10-day QuintSHIME^®^ experiment. The optimized enzyme mix improved the digestibility of FODMAP in the diet, demonstrating the digestion of lactose, fructans, and galactans, and reduced an undesirable increase in gas pressure. Additionally, the optimized enzyme mix restored the loss of microbial diversity observed with 100% FODMAP. However, a reduction in both SCFA production and absolute abundance of Firmicutes, Bacteroidetes, *Bifidobacterium*, and *F. prausnitzii* also indicated that certain microbial activity is not stimulated by the optimized enzyme mix in contrast to undigested FODMAP. HMO co-supplementation compensated for the loss of growth of beneficial microbes and their metabolic activities, demonstrated by increased SCFA levels and abundance of beneficial microbes (Firmicutes and *Lachnospiraceae* [including *E. ramulus*, *C. bolteae*, *B. obeum/wexlerae* and *Lachnospiraceae* spp.]) and decreased absolute abundance of detrimental microbes (Desulfobacteria, *Desulfovibrionaceae*). However, these beneficial effects on the gut microbiome were paralleled by increases in gas production. Overall, the addition of HMO was, therefore, effective at rebalancing gut microbiota composition of the FODMAP ingredients in the presence of FODMAP enzymes. 

When unabsorbed carbohydrates reach the colon, they attract water to the colon lumen and gas production is increased via colonic bacterial fermentation which together can lead to luminal distension [33]. In a crossover study of patients with inflammatory bowel disease who consumed a low-FODMAP diet and then a high-FODMAP diet, the high-FODMAP diet was significantly associated with an increase in abdominal pain/discomfort, abdominal bloating, and excessive flatulence when compared with the low-FODMAP diet [34]. These symptoms are likely associated with colonic gas production. In our study, FODMAP resulted in increased gas pressure relative to blank and when the optimized enzyme mix was added, gas pressure was significantly reduced. Although gas pressure was increased with pre-digested FODMAP + HMO relative to pre-digested FODMAP, it is important to note that it was decreased compared with FODMAP + HMO. It is possible that the increase in gas pressure with the addition of HMO in the case of pre-digested FODMAP may not prove to be an issue for patients with IBS, as HMOs have been shown to alleviate IBS symptoms when used as a standalone treatment [21,22]. 

Given the well-known health benefits of SCFAs (reviewed in [35]), a concern with the low-FODMAP diet is the potential for lower SCFA production by saccharolytic colonic bacteria. Our study indeed demonstrated an increase in total SCFA, acetate, propionate, and butyrate with FODMAP. However, the optimized enzyme mix reduced the level of total SCFA and most individual SCFAs to a level equivalent or similar to the blank. With pre-digested FODMAP + HMO, the levels were restored to or increased over those of FODMAP alone in most cases; in all cases, the SCFA levels were increased above pre-digested FODMAP. These results indicate that while the optimized enzyme mix reduces the beneficial effects of the FODMAP ingredients on microbiome composition and metabolic function, the addition of HMO can effectively restore this. 

A low-FODMAP diet has been associated with a six-fold reduction in Bifidobacteria in the stool of patients with IBS [36] though another study showed no significant differences in the absolute or relative abundance of Bifidobacteria [37]. In general, Bifidobacteria are considered beneficial to the human gut [38]. For example, administration of Bifidobacteria has been shown to reduce diarrhea, reduce the incidence of necrotizing entercolitis, to improve colon regularity, to prevent gastrointestinal infections via competitive exclusion of pathogens, and to reduce symptoms of inflammatory bowel disease. Prebiotic benefits are often measured based on changes in the abundance of Bifidobacteria. Although the biological consequences of reduced Bifidobacteria levels in patients with IBS are unknown, restoring their levels may be beneficial. In the short-term colonic batch experiments, the Bifidobacteria levels were numerically the highest with pre-digested FODMAP + HMO compared with the other treatments. Firmicutes levels were also numerically highest with pre-digested FODMAP + HMO compared with the other treatments. Several studies have reported a reduction of the butyrate producing *F. prausnitzii*, an important member of the Firmicutes genera, in patients with IBS [39] and a small study reported that a low-FODMAP diet reduced *F. prausnitzii* in patients with IBS [16]. Our study demonstrated that FODMAP alone and pre-digested FODMAP + HMO resulted in somewhat increased levels of *F. prausnitzii* in the fecal microbiota of patients with IBS. This could be considered a potential health benefit, as *F. prausnitzii* is reported to exert anti-inflammatory and protective effects in the gut and low levels of *F. prausnitzii* are thought to have a negative effect on the integrity of the intestinal mucous barrier [39,40]. The Firmicutes/Bacteroidetes ratio is an important indicator of normal intestinal homeostasis, with a decrease in this ratio being associated with inflammatory bowel disease [41]. In our study, pre-digested FODMAP + HMO resulted in an increased Firmicutes/Bacteroidetes ratio compared with pre-digested FODMAP, providing evidence of the prebiotic and beneficial effect of HMO. 

During the QuintSHIME^®^ experiments, *Bifidobacteriaceae* levels increased versus control with both donors after FODMAP supplementation, indicating that *Bifidobacteriaceae* were involved in primary substrate degradation of FODMAP compounds. This increase was not observed with pre-digested FODMAP; HMO co-supplementation resulted in a slight increase in *Bifidobacteriaciae* versus blank for one donor. For both donors, *Lachnospiraceae* were increased with HMO supplementation regardless of FODMAP digestion. The *Lachnospiraceae* family includes many butyrate producers; levels of this family are reportedly reduced in patients with Crohn’s disease and ulcerative colitis [42,43]. Supplementation with 100% FODMAP resulted in an increase in *Ruminococcaceae*, which include important butyrate-producing organisms. Pre-digested FODMAP counteracted a reduction in butyrate-producing *Erysipelotrichaceae* and acetate-producing *Acidaminococcaceae* levels observed with 100% FODMAP. This result indicates that pre-digestion of FODMAP with the optimized enzyme mix and co-supplementation with HMO may have positive effects on the microbial community composition by promoting the growth of important SCFA-producing families. Finally, pre-digestion of FODMAP, both with and without HMO, resulted in a Simpson Diversity Index value similar to that seen with the blank, while FODMAP addition, with and without HMO, resulted in significantly lower Simpson Diversity Index values compared with blank, indicating that the optimized enzyme mix supports microbial diversity in the gut. We hypothesize that providing a high level of a specific nutrient, in our case, FODMAP, may select for the strong outgrowth of certain microbial groups, even in the presence of HMO. The nutrients available in the colon with undigested FODMAP are reduced with pre-digestion, thus, the growth selection observed with undigested FODMAP is no longer present and microbial diversity remains high in samples with pre-digested FODMAP. This hypothesis is supported by the observation that prebiotic supplementation can result in reduced bacterial diversity in the proximal colon, mainly due to stimulation of specific microbial groups at the first site of fermentation [44].

This study had some limitations. As with all in vitro studies, the results reported here cannot be directly translated to an in vivo biological response. Rather, this study has utilized in vitro approaches to evaluate an enzyme mix to aid in the digestion of FODMAP ingredients and to determine whether the addition of HMOs could compensate for the depleted effect of the enzyme mix alone using microbiota isolated from patients with IBS-D. These findings provide a basis for future studies of the effects of an enzyme mix with or without HMO in patients with IBS. Additionally, our study identified that responses may vary with the donor microbiome, demonstrating that the findings are limited based on the small number of donors used in this study. Further work with a higher number of donors may shed more light on the response to the treatments in terms of interindividual variability. 

## 5. Conclusions

Our study identified an optimized enzyme mix to aid in the digestion of FODMAP ingredients. Using in vitro fermentation models with fecal inocula from patients with IBS-D, the optimized enzyme mix improved the digestibility of these ingredients and restored microbial diversity which was decreased with undigested FODMAP. This was accompanied by a reduction in the abundance of beneficial microbes, as well as reduced gas pressure and lower SCFA levels, which both indicate reduced microbial activity. The addition of HMO to the optimized enzyme mix restored these effects on the gut microbiota to that observed with undigested FODMAP, which was demonstrated by increases in SCFA and gas production (i.e., increased microbial activity) and an increase in *Lachnospiraceae* abundance, while maintaining microbial diversity. When used in combination, the optimized enzyme mix and HMO may improve the digestion of FODMAP ingredients while retaining their beneficial effects on microbiome composition and metabolic function. However, human intervention studies are now warranted to substantiate these findings and confirm that the increased gas production following HMO co-supplementation would not be a contributing factor to intestinal discomfort in IBS patients.

## Figures and Tables

**Table 1:** FODMAP digestion by individual FODMAP enzymes.

	Digestion Rate			
FODMAP Enzyme	FOS	Stachyose	Raffinose	Lactose
Maxinvert				
500 SU	46%	–	–	–
2500 SU	100%	–	–	–
10,000 SU	100%	–	–	–
Fibractase				
600 GalU	–	55%	24%	–
1200 GalU	–	63%	31%	–
2400 GalU	–	76%	52%	–
Tolerase L				
1000 ALU	–	–	–	56%
2500 ALU	–	–	–	76%
10,000 ALU	–	–	–	94%

FODMAP = fermentable oligosaccharides, disaccharides, monosaccharides, and polyols; FOS = fructooligosaccharides.

**Table 2:** Microbial diversity in the transverse colon.

	Reciprocal Simpson Diversity Index				
Donor	Blank	FODMAP	Pre-Digested FODMAP	FODMAP + HMO	Pre-Digested FODMAP + HMO
Donor 3	13.0	3.2 *	11.5	5.3 *	10.2
Donor 4	6.5	3.7 *	6.0	4.0 *	5.4
Average	9.7	3.5	8.7	4.7 *	7.8

* *p* < 0.05 versus respective blank. FODMAP = fermentable oligosaccharides, disaccharides, monosaccharides, and polyols; HMO = human milk oligosaccharide.

## Data Availability

Data will be made available upon acceptance.

## Supplementary Materials

The following supporting information can be downloaded at: https://www.mdpi.com/article/10.3390/nu15071637/s1, Figure S1: Firmicutes/Bacteroidetes ratio in short-term colonic batch experiments (48 h); Figure S2: Absolute abundance at the phylum, family, and species level for donor 3 (a) and donor 4 (b); Figure S3: Heatmap of the microbial community composition on day 9/10 according to donor at the genus level in the QuintSHIME^®^ model.

## References

[1] Gibson P.R., Shepherd S.J. (2005). Personal view: Food for thought--western lifestyle and susceptibility to Crohn’s disease. The FODMAP hypothesis. Aliment. Pharmacol. Ther..

[2] Gibson P.R., Shepherd S.J. (2010). Evidence-based dietary management of functional gastrointestinal symptoms: The FODMAP approach. J. Gastroenterol. Hepatol..

[3] Murray K., Wilkinson-Smith V., Hoad C., Costigan C., Cox E., Lam C., Marciani L., Gowland P., Spiller R.C. (2014). Differential effects of FODMAPs (fermentable oligo-, di-, mono-saccharides and polyols) on small and large intestinal contents in healthy subjects shown by MRI. Am. J. Gastroenterol..

[4] Barrett J.S., Gearry R.B., Muir J.G., Irving P.M., Rose R., Rosella O., Haines M.L., Shepherd S.J., Gibson P.R. (2010). Dietary poorly absorbed, short-chain carbohydrates increase delivery of water and fermentable substrates to the proximal colon. Aliment. Pharmacol. Ther..

[5] Sperber A.D., Dumitrascu D., Fukudo S., Gerson C., Ghoshal U.C., Gwee K.A., Hungin A.P.S., Kang J.Y., Minhu C., Schmulson M. (2017). The global prevalence of IBS in adults remains elusive due to the heterogeneity of studies: A Rome Foundation working team literature review. Gut.

[6] Major G., Pritchard S., Murray K., Alappadan J.P., Hoad C.L., Marciani L., Gowland P., Spiller R. (2017). Colon hypersensitivity to distension, rather than excessive gas production, produces carbohydrate-related symptoms in individuals with irritable bowel syndrome. Gastroenterology.

[7] Gibson P.R., Halmos E.P., Muir J.G. (2020). Review article: FODMAPS, prebiotics and gut health-the FODMAP hypothesis revisited. Aliment. Pharmacol. Ther..

[8] Bellini M., Tonarelli S., Nagy A.G., Pancetti A., Costa F., Ricchiuti A., de Bortoli N., Mosca M., Marchi S., Rossi A. (2020). Low FODMAP diet: Evidence, doubts, and hopes. Nutrients.

[9] Lacy B.E., Pimentel M., Brenner D.M., Chey W.D., Keefer L.A., Long M.D., Moshiree B. (2021). ACG clinical guideline: Management of irritable bowel syndrome. Am. J. Gastroenterol..

[10] McKenzie Y.A., Bowyer R.K., Leach H., Gulia P., Horobin J., O’Sullivan N.A., Pettitt C., Reeves L.B., Seamark L., Williams M. (2016). British Dietetic Association systematic review and evidence-based practice guidelines for the dietary management of irritable bowel syndrome in adults (2016 update). J. Hum. Nutr. Diet..

[11] O’Keeffe M., Lomer M.C. (2017). Who should deliver the low FODMAP diet and what educational methods are optimal: A review. J. Gastroenterol. Hepatol..

[12] Barrett J.S. (2017). How to institute the low-FODMAP diet. J. Gastroenterol. Hepatol..

[13] Maagaard L., Ankersen D.V., Vegh Z., Burisch J., Jensen L., Pedersen N., Munkholm P. (2016). Follow-up of patients with functional bowel symptoms treated with a low FODMAP diet. World J. Gastroenterol..

[14] O’Keeffe M., Jansen C., Martin L., Williams M., Seamark L., Staudacher H.M., Irving P.M., Whelan K., Lomer M.C. (2018). Long-term impact of the low-FODMAP diet on gastrointestinal symptoms, dietary intake, patient acceptability, and healthcare utilization in irritable bowel syndrome. Neurogastroenterol. Motil..

[15] Gearry R.B., Irving P.M., Barrett J.S., Nathan D.M., Shepherd S.J., Gibson P.R. (2009). Reduction of dietary poorly absorbed short-chain carbohydrates (FODMAPs) improves abdominal symptoms in patients with inflammatory bowel disease-a pilot study. J. Crohns. Colitis..

[16] Halmos E.P., Christophersen C.T., Bird A.R., Shepherd S.J., Gibson P.R., Muir J.G. (2015). Diets that differ in their FODMAP content alter the colonic luminal microenvironment. Gut.

[17] Dieterich W., Schuppan D., Schink M., Schwappacher R., Wirtz S., Agaimy A., Neurath M.F., Zopf Y. (2019). Influence of low FODMAP and gluten-free diets on disease activity and intestinal microbiota in patients with non-celiac gluten sensitivity. Clin. Nutr..

[18] Ianiro G., Pecere S., Giorgio V., Gasbarrini A., Cammarota G. (2016). Digestive enzyme supplementation in gastrointestinal diseases. Curr. Drug. Metab..

[19] Musilova S., Rada V., Vlkova E., Bunesova V. (2014). Beneficial effects of human milk oligosaccharides on gut microbiota. Benef. Microbes..

[20] Suligoj T., Vigsnaes L.K., Abbeele P.V.D., Apostolou A., Karalis K., Savva G.M., McConnell B., Juge N. (2020). Effects of human milk oligosaccharides on the adult gut microbiota and barrier function. Nutrients.

[21] Palsson O.S., Peery A., Seitzberg D., Amundsen I.D., McConnell B., Simren M. (2020). Human milk oligosaccharides support normal bowel function and improve symptoms of irritable bowel syndrome: A multicenter, open-label trial. Clin. Transl. Gastroenterol..

[22] Iribarren C., Tornblom H., Aziz I., Magnusson M.K., Sundin J., Vigsnaes L.K., Amundsen I.D., McConnell B., Seitzberg D., Ohman L. (2020). Human milk oligosaccharide supplementation in irritable bowel syndrome patients: A parallel, randomized, double-blind, placebo-controlled study. Neurogastroenterol. Motil..

[23] Mackie A., Rigby N. (2015). InfoGest consensus method. The Impact of Food Bioactives on Health.

[24] Van den Abbeele P., Kamil A., Fleige L., Chung Y., De Chavez P., Marzorati M. (2018). Different oat ingredients stimulate specific microbial metabolites in the gut microbiome of three human individuals in vitro. ACS Omega.

[25] Molly K., Vande Woestyne M., Verstraete W. (1993). Development of a 5-step multi-chamber reactor as a simulation of the human intestinal microbial ecosystem. Appl. Microbiol. Biotechnol..

[26] Possemiers S., Verthe K., Uyttendaele S., Verstraete W. (2004). PCR-DGGE-based quantification of stability of the microbial community in a simulator of the human intestinal microbial ecosystem. FEMS Microbiol. Ecol..

[27] De Weirdt R., Possemiers S., Vermeulen G., Moerdijk-Poortvliet T.C., Boschker H.T., Verstraete W., Van de Wiele T. (2010). Human faecal microbiota display variable patterns of glycerol metabolism. FEMS Microbiol. Ecol..

[28] Van den Abbeele P., Verstrepen L., Ghyselinck J., Albers R., Marzorati M., Mercenier A. (2020). A novel non-digestible, carrot-derived polysaccharide (cRG-I) selectively modulates the human gut microbiota while promoting gut barrier integrity: An integrated in vitro approach. Nutrients.

[29] Sokol H., Seksik P., Furet J.P., Firmesse O., Nion-Larmurier I., Beaugerie L., Cosnes J., Corthier G., Marteau P., Dore J. (2009). Low counts of Faecalibacterium prausnitzii in colitis microbiota. Inflamm. Bowel. Dis..

[30] Klindworth A., Pruesse E., Schweer T., Peplies J., Quast C., Horn M., Glockner F.O. (2013). Evaluation of general 16S ribosomal RNA gene PCR primers for classical and next-generation sequencing-based diversity studies. Nucleic. Acids Res..

[31] De Paepe K., Kerckhof F.M., Verspreet J., Courtin C.M., Van de Wiele T. (2017). Inter-individual differences determine the outcome of wheat bran colonization by the human gut microbiome. Environ. Microbiol..

[32] Van Nevel S., Koetzsch S., Weilenmann H.U., Boon N., Hammes F. (2013). Routine bacterial analysis with automated flow cytometry. J. Microbiol. Methods.

[33] Shepherd S.J., Lomer M.C., Gibson P.R. (2013). Short-chain carbohydrates and functional gastrointestinal disorders. Am. J. Gastroenterol..

[34] Ong D.K., Mitchell S.B., Barrett J.S., Shepherd S.J., Irving P.M., Biesiekierski J.R., Smith S., Gibson P.R., Muir J.G. (2010). Manipulation of dietary short chain carbohydrates alters the pattern of gas production and genesis of symptoms in irritable bowel syndrome. J. Gastroenterol. Hepatol..

[35] Gill P.A., van Zelm M.C., Muir J.G., Gibson P.R. (2018). Review article: Short chain fatty acids as potential therapeutic agents in human gastrointestinal and inflammatory disorders. Aliment. Pharmacol. Ther..

[36] Staudacher H.M., Lomer M.C., Anderson J.L., Barrett J.S., Muir J.G., Irving P.M., Whelan K. (2012). Fermentable carbohydrate restriction reduces luminal bifidobacteria and gastrointestinal symptoms in patients with irritable bowel syndrome. J. Nutr..

[37] Halmos E.P., Christophersen C.T., Bird A.R., Shepherd S.J., Muir J.G., Gibson P.R. (2016). Consistent prebiotic effect on gut microbiota with altered FODMAP intake in patients with Crohn’s disease: A randomised, controlled cross-over trial of well-defined diets. Clin. Transl. Gastroenterol..

[38] O’Callaghan A., van Sinderen D. (2016). Bifidobacteria and their role as members of the human gut microbiota. Front. Microbiol..

[39] Cao Y., Shen J., Ran Z.H. (2014). Association between Faecalibacterium prausnitzii reduction and inflammatory bowel disease: A meta-analysis and systematic review of the literature. Gatroenterol. Res. Pract..

[40] Sokol H., Pigneur B., Watterlot L., Lakhdari O., Bermudez-Humaran L.G., Gratadoux J.J., Blugeon S., Bridonneau C., Furet J.P., Corthier G. (2008). Faecalibacterium prausnitzii is an anti-inflammatory commensal bacterium identified by gut microbiota analysis of Crohn disease patients. Proc. Natl. Acad. Sci. USA.

[41] Stojanov S., Berlec A., Strukelj B. (2020). The influence of probiotics on the Firmicutes/Bacteroidetes ratio in the treatment of obesity and inflammatory bowel disease. Microorganisms.

[42] Vacca M., Celano G., Calabrese F.M., Portincasa P., Gobbetti M., De Angelis M. (2020). The controversial role of human gut Lachnospiraceae. Microorganisms.

[43] Sasaki K., Inoue J., Sasaki D., Hoshi N., Shirai T., Fukuda I., Azuma T., Kondo A., Osawa R. (2019). Construction of a model culture system of human colonic microbiota to detect decreased Lachnospiraceae abundance and butyrogenesis in the feces of ulcerative colitis patients. Biotechnol. J..

[44] Zhang C., Yin A., Li H., Wang R., Wu G., Shen J., Zhang M., Wang L., Hou Y., Ouyang H. (2015). Dietary modulation of gut microbiota contributes to alleviation of both genetic and simple obesity in children. eBioMedicine.

