# Supplementary material for: In Vitro Effect of Enzymes and Human Milk Oligosaccharides on FODMAP Digestion and Fecal Microbiota Composition
Source: Nutrients. 2023 Mar 28;15(7):1637. doi: 10.3390/nu15071637 (PMC10097142; doi:10.3390/nu15071637)
Supplement: Supplementary file 1 [file nutrients-15-01637-s001.zip › nutrients-2148953-SI.pdf]

Supplementary Materials

In Vitro Effect of Enzymes and Human Milk Oligosaccharides on FODMAP Digestion and Fecal Microbiota Composition

Van T. Pham, Robert E. Steinert, Cindy Duysburgh, Jonas Ghyselinck, Massimo Marzorati

and Peter J. T. Dekker

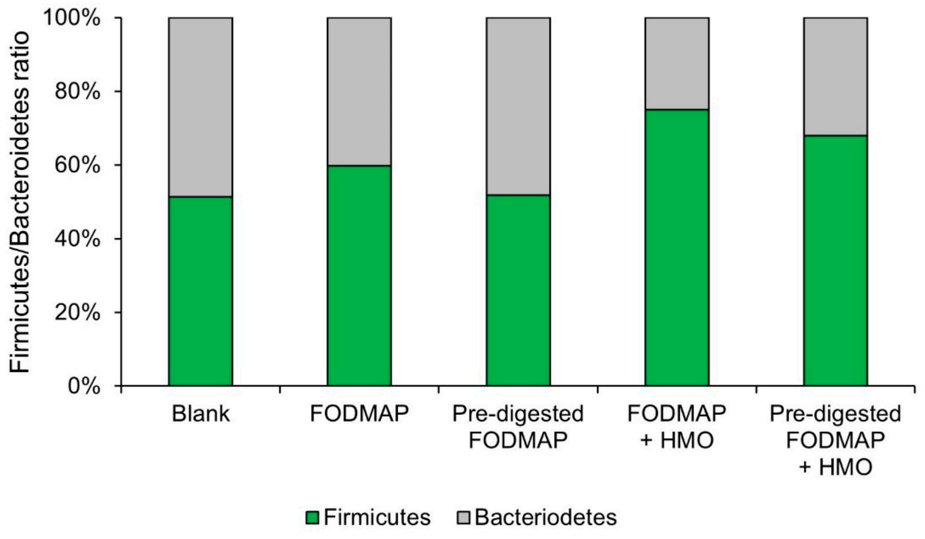

Figure S1. Firmicutes/Bacteroidetes ratio in short-term colonic batch experiments (48 hours). The test substrates were as follows: FODMAP (0.86 g/L FOS + 0.21 g/L stachyose + 0.21 g/L raffinose + 1.71 g/L lactose), pre-digested FODMAP (0.05 g/L stachyose + 0.10 g/L raffinose + 0.11 g/L lactose), FODMAP + HMO (FODMAP composition + 2.4 g/L 2'FL/DFL + 0.6 g/L lacto-N-neotetraose), pre-digested FODMAP + HMO (pre-digested FODMAP composition + 2.4 g/L 2'FL/DFL + 0.6 g/L lacto-N-neotetraose). Different letters represent statistically significant differences between the different test conditions ($p<0.05$). 2'FL = 2'fucosyllactose; DFL = difucosyllactose; FODMAP = fermentable oligosaccharides, disaccharides, monosaccharides and polyols; HMO = human milk oligosaccharide.

(a)

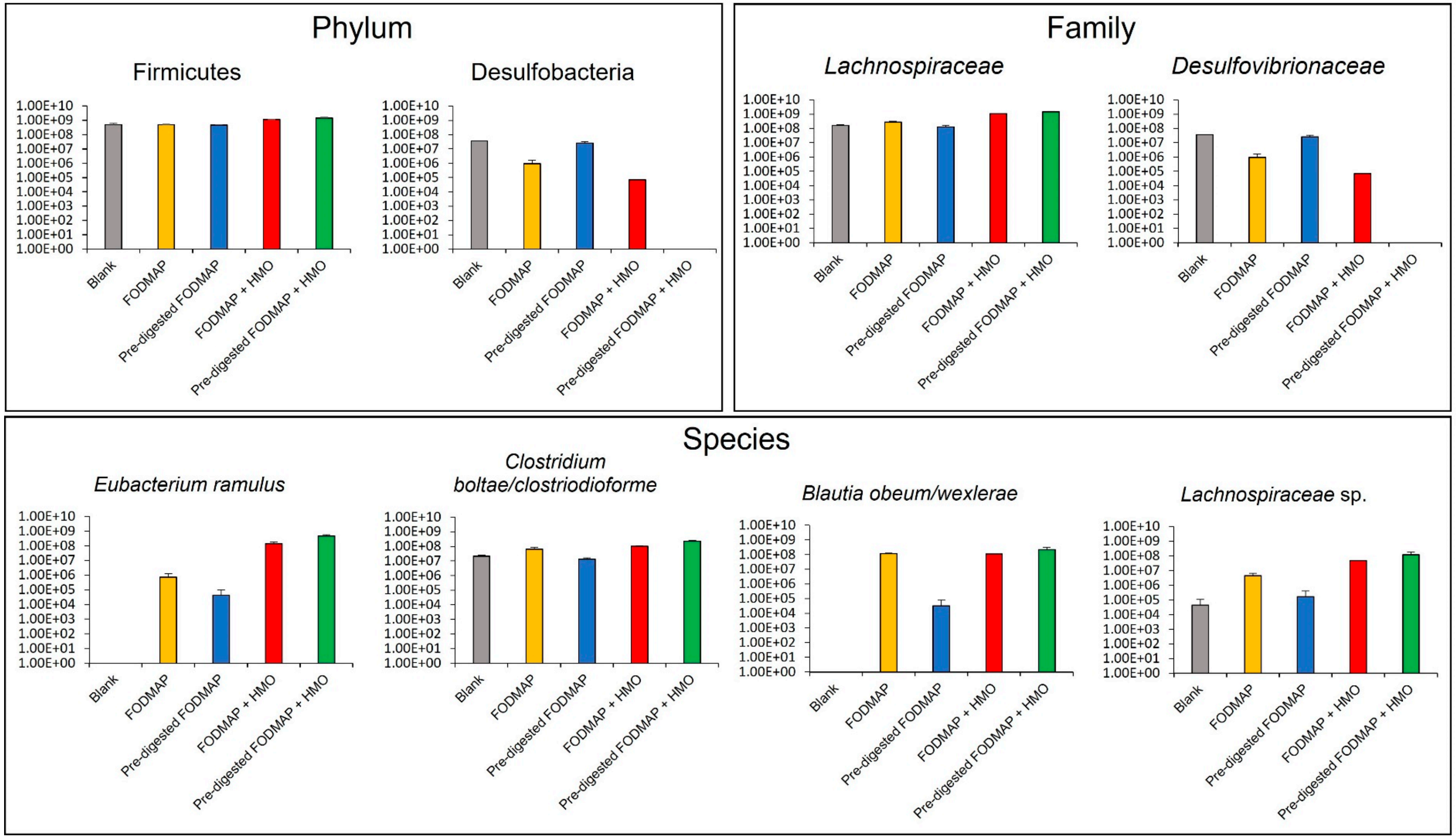

Phylum
Firmicutes
Desulfobacteria
Family
Lachnospiraceae
Desulfovibrionaceae
Species
Eubacterium ramulus
Clostridium boltae/clostridioforme
Blautia obeum/wexlerae
Lachnospiraceae sp.
1.00E+10
1.00E+09
1.00E+08
1.00E+07
1.00E+06
1.00E+05
1.00E+04
1.00E+03
1.00E+02
1.00E+01
1.00E+00
Blank
FODMAP
Pre-digested FODMAP
FODMAP + HMO
Pre-digested FODMAP + HMO

(b)

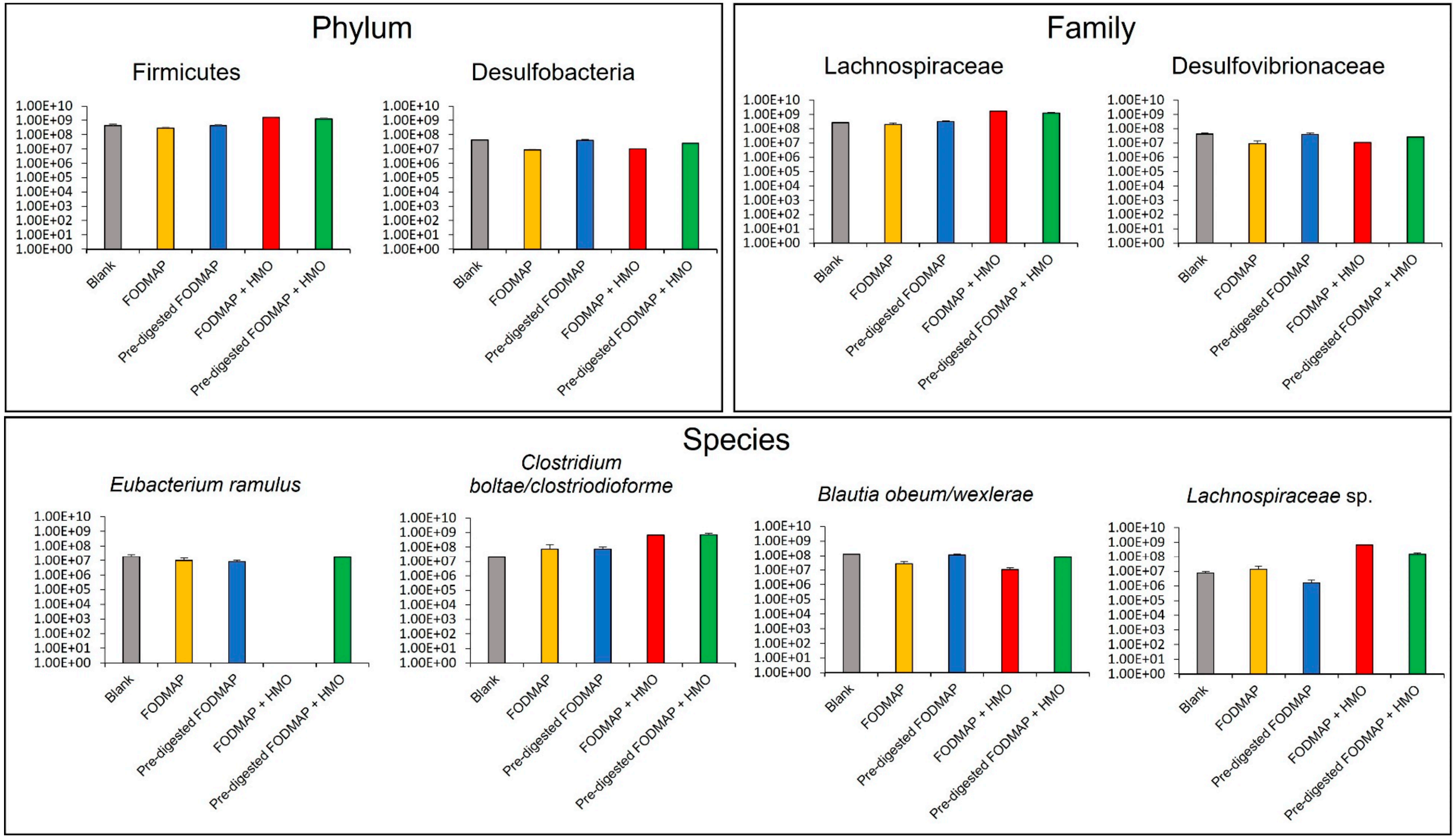
Phylum
Firmicutes
Desulfobacteria
Family
Lachnospiraceae
Desulfovibrionaceae
Species
Eubacterium ramulus
Clostridium boltae/clostridioforme
Blautia obeum/wexlerae
Lachnospiraceae sp.
1.00E+10
1.00E+09
1.00E+08
1.00E+07
1.00E+06
1.00E+05
1.00E+04
1.00E+03
1.00E+02
1.00E+01
1.00E+00
Blank
FODMAP
Pre-digested FODMAP
FODMAP + HMO
Pre-digested FODMAP + HMO

Figure S2. Absolute abundance at the phylum, family, and species level for donor 3 **(a)** and donor 4 **(b)**. FODMAP = fermentable oligosaccharides, disaccharides, monosaccharides and polyols; HMO = human milk oligosaccharide.

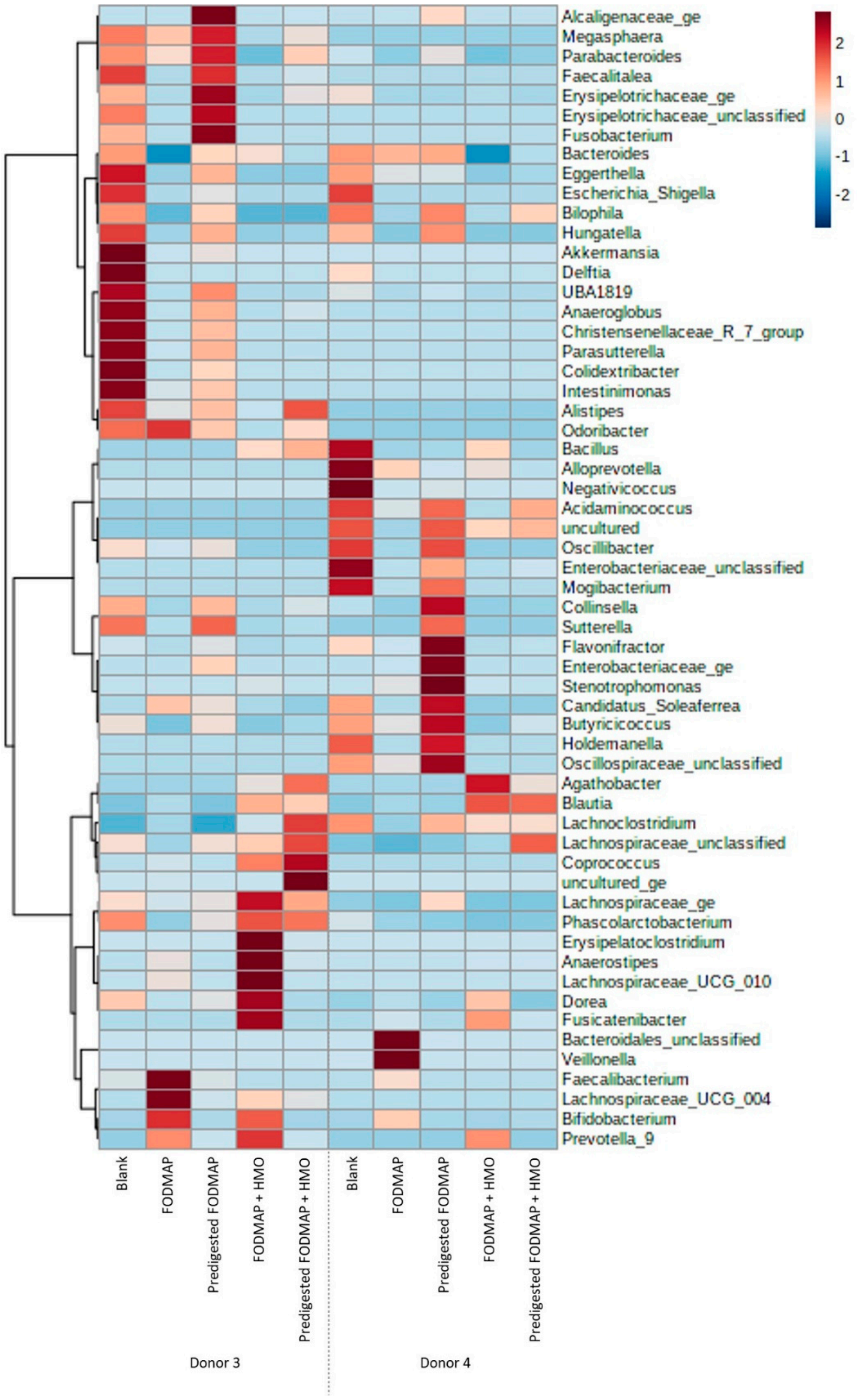

Figure S3. Heatmap of the microbial community composition on day 9/10 according to donor at the genus level in the QuintSHIME® model. TC samples collected on day 9 and day 10 were considered replicates. FODMAP = fermentable oligosaccharides, disaccharides, monosaccharides and polyols; HMO = human milk oligosaccharide; SHIME = simulator of the human intestinal microbial ecosystem; TC = transverse colon.
